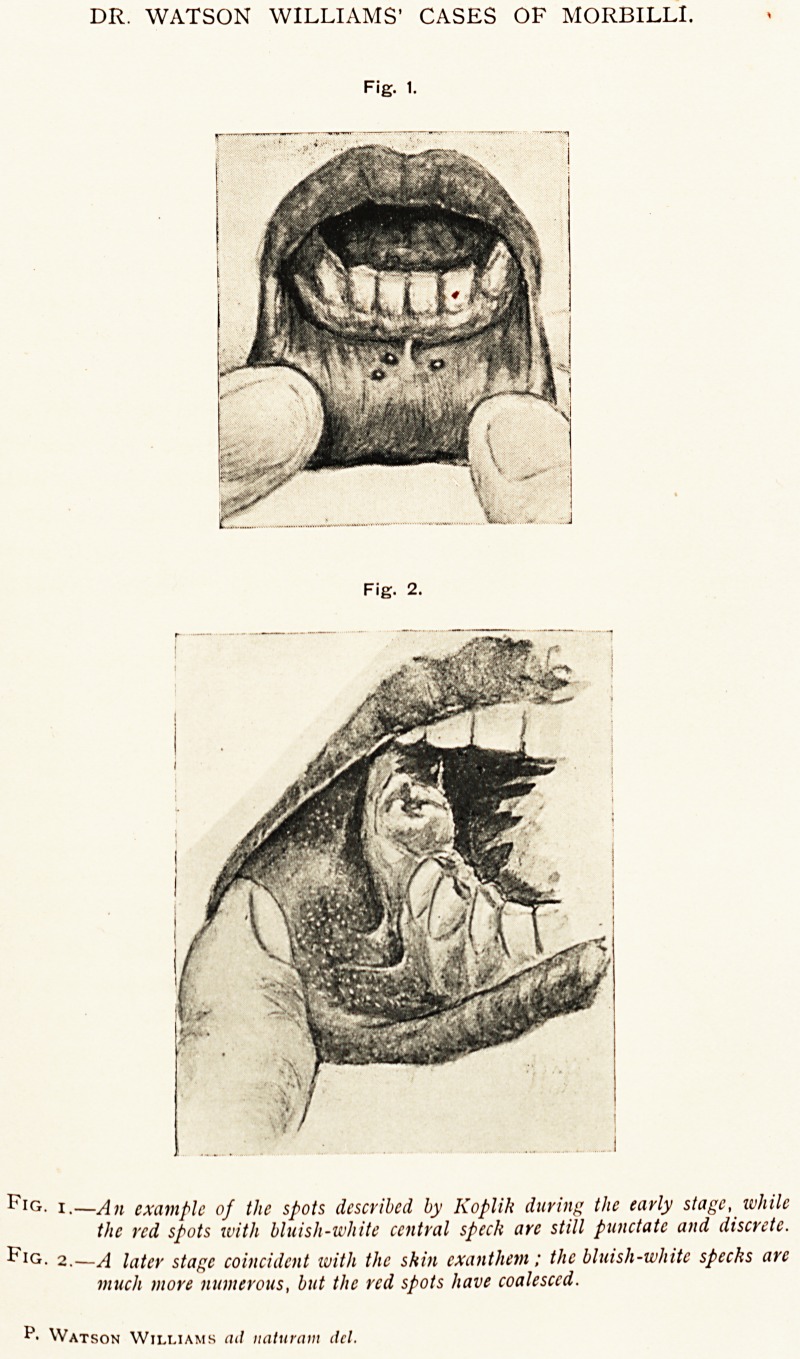# Note on a Pre-Exanthematous Sign of Measles

**Published:** 1900-06

**Authors:** P. Watson Williams

**Affiliations:** Physician to Clifton College; Physician in charge of the Department for Throat Diseases, Bristol Royal Infirmary, &c.


### NOTE ON A PRE-EXANTHEMATOUS SIGN OF MEASLES.

BY

P. WATSON WILLIAMS, M.D. Lond., &c.,

*Physician to Clifton College;*

*Physician in charge of the Department for Throat Diseases, Bristol Royal Infirmary, &c.*

---

THE pre-eruptive sign of measles described by Koplik, of New York, in 1896 as invariably present at some time in all cases of measles, and as absolutely pathognomonic of the affection, is worthy of closer attention than has been accorded to it in this country. The complete early isolation of patients developing measles is most desirable, inasmuch as the affection is highly contagious in the pre-eruptive stage, yet often the character of the symptoms that precede the appearance of the exanthem are so indefinite as to arouse no suspicion of commencing measles.

If the claims of Koplik that these spots are characteristic of measles are well founded, we have at hand a ready means of making a diagnosis before the appearance of the measles rash.

Moreover, there are various skin affections which closely simulate the rash of measles; and here again the sign now known as "Koplik's spots" may afford valuable data in making a differential diagnosis. The spots are very minute, round, discrete, bluish-white specks on a reddish punctate area or diffuse red background.

Koplik [1] states that "if we look in the mouth at this period [the time of the prodromal symptoms] we see a redness of the fauces; perhaps, not in all cases, a few spots on the soft palate. On the buccal mucous membrane and the inside of the lips, we invariably see a distinct eruption. It consists of small, irregular spots, of a bright red color. In the centre of each spot, there is noted, in strong daylight, a minute bluish white speck. These red spots, with accompanying specks of a bluish white color,

[1] *Arch. Pediat.*, 1896, xiii. 918.

are absolutely pathognomonic of beginning measles, and when seen can be relied upon as the forerunner of the skin eruption."

Once seen, they can hardly be mistaken. They vary in number from two or three to countless numbers, and are most usually seen on the portions of the buccal and labial mucosa in contact with the teeth. In looking for them the lips should be well everted, and the cheeks drawn away from the teeth first on one side and then on the other, by the tip of the finger or by the handle of a spoon. Strong daylight is better than artificial light. In many cases no distinct red spots are to be seen, but the white specks look like particles of salt lying on the surface of the reddened mucous membrane. These white spots are adherent, but may be rubbed off, leaving a smooth pink surface.

When occurring in crowds, although the spots do not actually coalesce, they may give at first sight the appearance of a thin white glazing or false membrane. The buccal mucous membrane, not that of the palate, is the place where their presence should be sought.

These spots appear from twelve hours to three days before the skin exanthem, but cases have been known on which the spots were present as much as five days before the rash. They generally begin to fade as the skin eruption becomes well developed.

I have found this sign of great value in arriving at an early diagnosis of measles, but I am unable to say from personal experience whether these spots are absolutely pathognomonic of measles. Koplik says that cases with the usual morbilliform eruption without these spots are really cases of rötheln. Ross,[1] who has made a special study of these spots in Koplik's clinic, states that in some of the children suffering from rötheln "there was everything resembling measles in the examination of the skin. The children were covered with an eruption which many physicians would have called undoubted cases of measles. The Koplik's spots were absent in these cases, and the throat nearly normal. In almost all cases the temperature was 100° to 101°. Not only is rötheln a distinct disease from measles, but the Koplik's spots

[1] *Columbus M. J.*, 1900, xix. 64.

DR. WATSON WILLIAMS' CASES OF MORBILLI.

Fig. 1.

Fig. 2.

FIG. 1.—*An example of the spots described by Koplik during the early stage, while the red spots with bluish-white central speck are still punctate and discrete.*

FIG. 2.—*A later stage coincident with the skin exanthem; the bluish-white specks are much more numerous, but the red spots have coalesced.*

P. WATSON WILLIAMS *ad naturam del.*

are the only, and most valuable, means of differentiating these from true measles. This may account for many of the cases of measles without Koplik's spots. He (Koplik) maintains that such cases are really pronounced cases of rötheln." I may add that I have diagnosed such cases of measles without Koplik's spots, but I am not prepared to admit that they were cases of rötheln. Nevertheless I am convinced that when Koplik's spots are present they invariably indicate the existence of morbilli.

Sobel,[1] who has observed the characteristic spots in numerous cases of measles, has particularly examined the buccal mucous membrane of children affected with various skin eruptions—varicella, urticaria, scarlatina, vaccinia, purpura simplex and hæmorrhagica, congenital syphilis, erythema multiforme, scabies, miliaria, eczema, rötheln, impetigo simplex and contagiosa, drug eruptions (bromides, antipyrine)—and in no case were Koplik's spots observed.

## Figures and Tables

**Fig. 1. Fig. 2. f1:**